# Supplementary material for: Violent video game engagement is not associated with adolescents' aggressive behaviour: evidence from a registered report
Source: R Soc Open Sci. 2019 Feb 13;6(2):171474. doi: 10.1098/rsos.171474 (PMC6408382; doi:10.1098/rsos.171474)
Supplement: Appendices detailing the self-report measures used in the study. [file rsos171474supp1.docx]

Appendix A*

**Instructions**

For each item, please mark the box for Not True, Somewhat True or Certainly True. It would help us if you answered all items as best you can even if you are not absolutely certain or the item seems daft! Please give your answers on the basis of your child's behaviour over the last month.

**Response Options**

0 | Not true

1 | Somewhat true

2 | Certainly true

**Response Items**

1. Considerate of other people's feelings
2. Restless, overactive, cannot stay still for long
3. Often complains of headaches, stomach-aches or sickness
4. Shares readily with other children (treats, toys, pencils etc.)
5. Often has temper tantrums or hot tempers
6. Rather solitary, tends to play alone
7. Generally obedient, usually does what adults request
8. Many worries, often seems worried
9. Helpful if someone is hurt, upset or feeling ill
10. Constantly fidgeting or squirming
11. Has at least one good friend
12. Often fights with other children or bullies them
13. Often unhappy, down-hearted or tearful
14. Generally liked by other children
15. Easily distracted, concentration wanders
16. Nervous or clingy in new situations, easily loses confidence
17. Kind to younger children
18. Often lies or cheats
19. Picked on or bullied by other children
20. Often volunteers to help others (parents, teachers, other children)
21. Thinks things out before acting
22. Steals from home, school or elsewhere
23. Gets on better with adults than with other children
24. Many fears, easily scared
25. Sees tasks through to the end, good attention span

*Note: outcome variable measure, completed by caregivers. Individual aggressive behaviour scores were computed for each participant. This was be done by calculating individuals’ scores on the conduct problems subscale of the SDQ (see Appendix C). Summing responses to items 5,7 (reverse coded),12,18, & 22). Each participant will therefore have a aggressive behaviour score that ranges from 5 to 15.

Appendix B**

**Instructions (item 1)**

Many young people regularly play video games on computers (e.g. Minecraft), smartphones (e.g. Candy Crush), and gaming consoles (e.g. New Super Mario Bros.). Do you play video games?

**Response Options**

0 | No

1 | Yes

**Instructions (item 2)** [show if yes]

Please name the games you played most in the past month and please try to be specific. For example, instead of typing “Mario Kart” or “COD” please type the specific game name, for example “Mario Kart 8” or “Call of Duty: Black Ops: Declassified”.

**Response Options**

- [open text]
- [open text]
- [open text]

**Instructions (item 3)** [for adolescent] (repeat for every game named, pipe in [game name])

How do you play [named game]?

**Response Option** [select one]

- Personal Computer (e.g. PC, MAC, laptop, etc.)
- Smartphone (e.g. iPhone, Andriod, etc.)
- Console (e.g. PlayStation, Xbox, Nintendo Wii, etc.)

**Instructions (item 4)** [for adolescent] (repeat for every game named, pipe in [game name])

Do you play this game with other people [named game]?

**Response Option** [select one]

- No
- Yes, online only
- Yes, offline only
- Yes, offline and online

**Note: part of explanatory measure, completed by adolescents.

Appendix C**

**Instructions** (repeat for every game named, pipe in [game name])

About how many hours a day do you usually play [named game] on in your free time?

**Response Options**

0 | None at all

.5 | About half an hour a day

1 | About 1 hour a day

2 | About 2 hours a day

3 | About 3 hours a day

4 | About 4 hours a day

5 | About 5 hours a day

6 | About 6 hours a day

7 | About 7 or more hours a day

**Note: part of explanatory measure, completed by adolescents.

Appendix D**

**Instructions**

Using the 5-point scale shown below, please indicate how uncharacteristic or characteristic each of the following statements are, in terms of describing you.

**Response Options**

1 | very unlike me

2 | unlike me

3 | neither like or unlike me

4 | like me

5 | very like me

**Response Items**

1. Given enough provocation, I may hit another person. (P)
2. I often find myself disagreeing with people. (V)
3. At times I feel I have gotten a raw deal out of life. (H)
4. There are people who have pushed me so far that we have come to blows. (P)
5. I can’t help getting into arguments when people disagree with me. (V)
6. Sometimes I fly off the handle for no good reason. (A)
7. Other people always seem to get the breaks. (H)
8. I have threatened people I know. (P)
9. My friends say that I’m somewhat argumentative. (V)
10. I have trouble controlling my temper. (P)
11. I wonder why sometimes I feel so bitter about things. (H)
12. I sometimes feel like a powder keg ready to explode. (A)

P = physical; V = verbal; A = anger; H = hostility.

**Note: control variable measure, completed by adolescents. Individual P = physical; V = verbal; A = anger; H = hostility scores will be computed for each participant by averaging responses to items in each subscale.

Appendix E**

**Instructions**

Using the 5-point scale shown below, please indicate how much you agree with the following statement.

**Response Options**

1 | Strongly disagree

2 | Disagree

3 | Neither agree or disagree

4 | Agree

5 | Strongly agree

**Response Items**

I spend a lot of time playing video games.

**Note: a check measure, completed by adolescents.
